# Efficacy and biomarker analysis for nivolumab in cancer of unknown primary: results from an investigator-initiated expanded access program

**DOI:** 10.1007/s10147-026-03187-9

**Published:** 2026-09-24

**Authors:** Junko Tanizaki, Kan Yonemori, Yuichi Takiguchi, Kohei Akiyoshi, Keigo Komine, Yusuke Onozawa, Mariko Sato, Kenro Hirata, Takuma Onoe, Ryotaro Ohkuma, Yosuke Horita, Hironobu Minami, Kazunori Honda, Koichi Suyama, Akihiko Ito, Yasutaka Chiba, Hiroko Watanabe, Yosuke Togashi, Kazuko Sakai, Kazuto Nishio, Kazuhiko Nakagawa, Hidetoshi Hayashi

**Affiliations:** 1 Department of Medical Oncology, Kindai University Faculty of Medicine, 1-14-1 Mihara-dai, Minami-ku, Sakai, Osaka 590-0197 Japan; 2 Department of Medical Oncology, National Cancer Center Hospital, Tokyo, Japan; 3 Medical Oncology Department, Chiba University, Chiba, Japan; 4 Department of Medical Oncology, Osaka City General Hospital, Osaka, Japan; 5 Department of Medical Oncology, Tohoku University Hospital, Sendai, Japan; 6 Division of Clinical Oncology, Shizuoka Cancer Center, Shizuoka, Japan; 7 Department of Medical Oncology, NHO Nagoya Medical Center, Nagoya, Japan; 8 Division of Gastroenterology and Hepatology, Department of Internal Medicine, Keio University School of Medicine, Tokyo, Japan; 9 Department of Medical Oncology, Hyogo Cancer Center, Akashi, Japan; 10 Department of Medicine, Division of Medical Oncology, Graduate School of Medicine, Showa Medical University, Tokyo, Japan; 11 Department of Medical Oncology, Saitama Medical University International Medical Center, Saitama, Japan; 12 Department of Medical Oncology/Hematology, Kobe University Graduate School of Medicine and Hospital, Kobe, Japan; 13 Department of Clinical Oncology, Aichi Cancer Center Hospital, Nagoya, Japan; 14 Department of Medical Oncology, Toranomon Hospital, Tokyo, Japan; 15 Department of Pathology, Kindai University Faculty of Medicine, Osaka, Japan; 16 Clinical Research Center, Kindai University Hospital, Osaka, Japan; 17 Department of Tumor Microenvironment, Faculty of Medicine, Okayama University Graduate School of Medicine, Dentistry, and Pharmaceutical Sciences, Okayama, Japan; 18 Department of Genome Biology, Kindai University Faculty of Medicine, Osaka, Japan; 19 Kindai University Hospital Cancer Center, Osaka, Japan

**Keywords:** Nivolumab, Cancer of unknown primary (CUP), Expanded access program

## Abstract

**Background:**

Cancer of unknown primary (CUP) remains a challenging malignancy with a poor prognosis. Although immune checkpoint inhibitors have shown activity in CUP, available data remains limited. We describe clinical outcomes and a comprehensive biomarker analysis from an expanded access trial of nivolumab in CUP.

**Methods:**

Patients with CUP were enrolled regardless of treatment history and received intravenous nivolumab (240 mg every 2 weeks or 480 mg every 4 weeks) until disease progression, unacceptable toxicity, or withdrawal. Enrollment concluded after regulatory approval of nivolumab for CUP in Japan. The primary objective was safety; secondary endpoints addressed efficacy, and exploratory biomarker analyses were performed.

**Results:**

A total of 53 patients (31 previously treated, 22 untreated) received nivolumab. Adverse events (AEs) occurred in 77.4% of patients, including 24.5% grade 3–4 and 15.1% serious AEs; no treatment-related deaths occurred. The objective response rate was 17.0% (95% confidence interval [CI] 8.1–29.8%) overall and 20.5% (95% CI 9.8–35.3%) in the response-evaluable subset. Median progression-free survival was 4.5 months (95% CI 2.9–7.9) and median overall survival was 17.5 months (95% CI 11.9–25.1). Higher PD-L1 expression was associated with better efficacy, whereas no meaningful differences were observed across estimated tissue-of-origin subgroups.

**Conclusions:**

This study provides additional prospective evidence consistent with the preceding NivoCUP trial, supporting the reproducibility of the efficacy and safety of nivolumab for patients with CUP. These findings support PD-1-targeted therapy as a feasible treatment option for this rare malignancy.

**Trial registration:**

Japan Registry of Clinical Trials (jRCT) identifier, jRCT2051200146.

**Supplementary Information:**

The online version contains supplementary material available at https://doi.org/10.1007/s10147-026-03187-9.

## Introduction

Cancer of unknown primary (CUP) accounts for 2% to 5% of all malignancies [1, 2]. Despite recent diagnostic advances, the prognosis of individuals with CUP remains poor, with a median overall survival (OS) of only 6 to 12 months under conventional platinum-based chemotherapy [3–6]. This dismal outcome underscores the urgent need for more effective, biology-driven therapeutic strategies.

Recent therapeutic developments for CUP have been based on two distinct approaches: site-specific genomic profiling and tissue-agnostic immunotherapy. Although several trials have demonstrated the feasibility of genome-guided site-specific treatment [7–10], the development of immune checkpoint inhibitor (ICI) therapy has faced substantial obstacles. The investigator-initiated NivoCUP trial demonstrated the clinical activity of nivolumab in previously treated individuals with CUP, resulting in its landmark regulatory approval in Japan [11]. However, the clinical development of other ICIs for CUP has remained limited to small subcohorts of basket trials, such as those for pembrolizumab [12], or has been hampered by early termination of combination studies (such as for ipilimumab plus nivolumab) as a result of poor patient accrual [13]. Given this landscape, the provision of reproducible evidence from prospective clinical studies is essential for establishment of the true clinical benefit of ICIs for CUP.

We here present the results of an investigator-initiated expanded access program (EAP) of nivolumab, an antibody to programmed cell death-1 (PD-1), for individuals with CUP. We also performed comprehensive immunologic evaluations in this EAP to elucidate the relation between the tumor microenvironment and therapeutic response in this rare population.

## Patients and methods

### Study design, patients, and treatment

The study was designed as an investigator-initiated EAP of nivolumab in individuals with CUP (NivoCUP2, WJOG14620M; jRCT identifier, jRCT2051200146). The study protocol and eligibility criteria were based on a preceding pivotal phase II trial [11], with modifications to reflect real-world clinical practice. Specifically, patients were eligible regardless of the presence of measurable lesions and were allowed to have an Eastern Cooperative Oncology Group performance status of 0 to 2. Less restrictive organ function criteria (total bilirubin concentration of < 2.0 mg/dL, and serum creatinine concentration of < 2.0 mg/dL or creatinine clearance of ≥ 30 mL/min) were also applied. With the exception of these specified modifications, the eligibility requirements were identical to those of the pivotal trial, including mandatory examinations for diagnosis of CUP and consultations with specific departments. The protocol was approved by the institutional review board at each site, and the study was conducted in accordance with the provisions of the Declaration of Helsinki and the International Conference for Harmonisation Good Clinical Practice Guideline. All patients provided written informed consent before study entry.

All enrolled patients received intravenous nivolumab at a dose of 240 mg every 2 weeks or 480 mg every 4 weeks until disease progression, unacceptable toxicity, or study withdrawal. Participants were allowed to receive treatment beyond disease progression if they met prespecified criteria, as described in the preceding pivotal trial [11]. Per the protocol, study enrollment and treatment were scheduled to conclude on regulatory approval of the partial change in the marketing authorization of nivolumab for CUP in Japan.

For evaluation of efficacy outcomes beyond termination of the EAP, patients who were alive at the time of study completion and provided consent for a subsequent observational study (NivoCUP2F, WJOG14620MFS; UMIN identifier, UMIN000046500) were followed up. In this observational study, efficacy endpoints including objective response rate (ORR), disease control rate (DCR), progression-free survival (PFS), OS, and duration of response (DoR) were assessed. Eligibility for the observational study was restricted to patients who were alive at the closure of the EAP, and participation was based on written informed consent or, when an opportunity for direct explanation was not available, on an opt-out procedure in accordance with the Japanese ethical guidelines for medical and biological research involving human subjects. The observational study collected efficacy and survival data only, including the date of the first subsequent anticancer therapy and the date of death or of the last confirmation that the patient was alive; no further safety data were collected after the end of the EAP. Time-to-event endpoints were measured continuously from the date of the first dose of nivolumab administered in the EAP and were not restarted at entry into the observational study. Nivolumab that was continued as routine clinical care after the closure of the EAP was, in accordance with the protocol of the observational study, not regarded as subsequent anticancer therapy, and patients who continued such treatment were therefore not censored at that time point. Patients who did not participate in the observational study were censored at the date last known to be alive as of the closure of the EAP. The disposition of the patients is shown in Supplementary Figure S1.

### Endpoints and assessments

The primary objective of the present study was to evaluate the safety profile of nivolumab monotherapy in individuals with CUP. The primary endpoint was accordingly the incidence of adverse events (AEs). Secondary endpoints focused on efficacy were assessed according to Response Evaluation Criteria in Solid Tumors (RECIST) version 1.1 and included ORR, DCR, PFS, OS, DoR, and best overall response. Tumor responses were assessed by the investigators without independent central radiologic review, and confirmation of response was not required. As patients were eligible irrespective of the presence of measurable disease, the objective response rate and best overall response were evaluated in all treated patients (n = 53), whereas the best percentage change in target-lesion size could be determined only in the response-evaluable subset with at least one measurable lesion (n = 44). In addition, as an exploratory endpoint, the relation between the therapeutic efficacy of nivolumab and programmed cell death–ligand 1 (PD-L1) expression status was assessed by immunohistochemistry with cutoff values of 1%, 10%, and 50%.

During the EAP, radiologic tumor assessment was performed at baseline and thereafter on day 1 of cycle 3 and of every second cycle thereafter, corresponding to an interval of 8 weeks, and tumor response was assessed according to RECIST version 1.1. After the closure of the EAP, imaging during the observational study was performed at the discretion of the treating physician as part of routine clinical care, and no protocol-specified imaging schedule was applied. AEs were monitored throughout the study and for 28 days after discontinuation of study treatment, and they were graded by investigators according to the National Cancer Institute Common Terminology Criteria for Adverse Events (CTCAE) version 5.0.

### Statistical analysis

Sample size was determined on the basis of the feasibility and enrollment performance of the preceding NivoCUP study [11], which accrued 55 patients between February 2018 and August 2019. Given the consistent accrual rate, we set the target sample size for the present trial at 60 patients to examine the reproducibility of the findings of the primary study. The efficacy and safety analysis sets included all enrolled patients who received at least one dose of nivolumab. Kaplan–Meier survival curves were constructed for PFS and OS. Medians of time-to-event endpoints and their two-sided 95% CIs were estimated by the Kaplan–Meier method with the Brookmeyer–Crowley procedure. For PFS, disease progression was defined according to RECIST version 1.1, and clinical deterioration without radiologic progression was not counted as an event; patients without documented progression or death were censored at the date of the last evaluable tumor assessment, and those who began subsequent anticancer therapy before progression or death were censored at the last evaluable assessment before that therapy. For OS, patients not known to have died were censored at the date last known to be alive. Hazard ratios (HRs) and associated 95% confidence intervals (CIs) were calculated with Cox proportional hazards models (Wald method) for the subgroup comparisons of progression-free survival, overall survival, and duration of response. These Cox models were univariable, and the resulting hazard ratios are unadjusted and descriptive given the small subgroup sizes. Tumor responses were compared according to PD-L1 tumor proportion score (TPS) or combined positive score (CPS) at cutoffs of 1%, 10%, and 50%. Binomial 95% confidence intervals for response rates (objective response rate and disease control rate) were calculated using the Clopper–Pearson exact method. All biomarker analyses were exploratory, and no adjustment for multiple comparisons was applied. The immune-profiling comparisons between responders and patients with progressive disease were descriptive; no formal statistical testing was performed and no P values were calculated. Patients for whom a given biomarker sample was unavailable or inadequate were excluded from the corresponding analysis, and no imputation was performed. As prespecified in the protocol, safety endpoints were summarized descriptively as the numbers and proportions of patients with adverse events; neither confidence intervals nor formal hypothesis testing was prespecified for the safety endpoints. Statistical analysis was performed with SAS version 9.4 software (SAS Institute, Cary, NC, USA).

### Assessments of tumor characteristics and biomarker analysis

Comprehensive biomarker analysis was performed with tumor tissue and blood samples. The tumor primary site was predicted with the use of a previously validated algorithm [10, 11]. Details of other molecular and immunologic assessments, including PD-L1 immunohistochemistry (TPS and CPS); analysis of microsatellite instability (MSI) status, tumor mutation burden (TMB), and T-cell receptor (TCR) beta repertoire; and flow cytometry of peripheral blood mononuclear cells (PBMCs) are provided in the Supplementary Methods.

## Results

### Patients

Between May and December 2021, a total of 54 patients with CUP, including 31 previously treated and 23 previously untreated individuals, were enrolled in this EAP. Of these patients, 53 individuals received nivolumab treatment and were included in the efficacy and safety analyses, with one individual not receiving treatment and therefore being excluded. Patient and disease characteristics are summarized in Supplementary Table S1. Among the previously treated patients, more than half (54.8%) had received treatment with carboplatin plus paclitaxel (Supplementary Table S2). After the approval of nivolumab for CUP in Japan, the NivoCUP2 trial was concluded on 22 December 2021. At that time, patients who were undergoing treatment were allowed to continue nivolumab therapy through standard clinical practice, and their survival and treatment efficacy were monitored as part of an observational study, NivoCUP2F (WJOG14620MFS, UMIN000046500) (Supplementary Figure S1). Of the 53 treated patients, 48 were alive at the closure of the EAP, and 47 of these individuals were followed up in the observational study; no additional safety data were collected during this period.

### Safety

Among all patients, 41 (77.4%) experienced AEs of any grade, with 13 (24.5%) experiencing AEs of grade 3 or 4 (Table 1). Serious AEs were observed in 8 (15.1%) patients, whereas no individual discontinued nivolumab because of AEs. No treatment-related death occurred. AEs with a frequency of ≥ 10% were pyrexia (n = 7, 13.2%), rash (n = 7, 13.2%), and elevated aspartate aminotransferase (AST) (n = 6, 11.3%). Immune-related AEs occurred in 15 (28.3%) of the 53 patients, with all these events being grade 1 or 2. No new safety signals were identified. Treatment-related AEs occurred in 25 (47.2%) patients, of which one event was grade 3 and serious (colitis). Systemic corticosteroid therapy for an immune-related event was required in only one patient (prednisolone for grade 2 diarrhea). The median duration of nivolumab exposure was 70 days (range, 1–197 days).

**Table 1:** Incidence of adverse events (AEs) for all patients (*n* = 53)

	Overall, *n* (%) [95% CI]
All AEs	41 (77.4) [63.8–87.7]
Grade 3 or 4 AEs	13 (24.5) [13.8–38.3]
Serious AEs	8 (15.1) [6.7–27.6]
Treatment-related AEs	25 (47.2) [33.3–61.4]
Grade ≥ 3 treatment-related AEs	1 (1.9) [0.0–10.1]
AEs leading to treatment discontinuation	0 (0.0) [0.0–6.7]
Treatment-related AEs leading to death	0 (0.0) [0.0–6.7]

	Any grade, *n* (%)	Grade 3 or 4
AEs with a frequency of ≥ 10%		
Pyrexia	7 (13.2)	0 (0.0)
Rash	7 (13.2)	0 (0.0)
AST increased	6 (11.3)	1 (1.9)
Immune-related AEs	15 (28.3)	0 (0.0)
Diarrhea/colitis		
Diarrhea	2 (3.8)	0 (0.0)
Hepatitis		
AST increased	1 (1.9)	0 (0.0)
ALT increased	1 (1.9)	0 (0.0)
Hepatic failure	1 (1.9)	0 (0.0)
Endocrine disorders		
Hyperthyroidism	1 (1.9)	0 (0.0)
Hypothyroidism	3 (5.7)	0 (0.0)
Adrenal insufficiency	2 (3.8)	0 (0.0)
Nephritis/urinary disorders		
Hemorrhagic cystitis	1 (1.9)	0 (0.0)
Rash		
Rash	6 (11.3)	0 (0.0)
Pruritus	3 (5.7)	0 (0.0)
Rash maculopapular	1 (1.9)	0 (0.0)
Dermatitis	1 (1.9)	0 (0.0)
Myositis		
CPK increased	1 (1.9)	0 (0.0)

ALT, alanine aminotransferase; AST, aspartate aminotransferase; CPK, creatine phosphokinase. Values in square brackets are post hoc two-sided 95% confidence intervals for the summary safety endpoints

### Efficacy

**Fig. 1:**
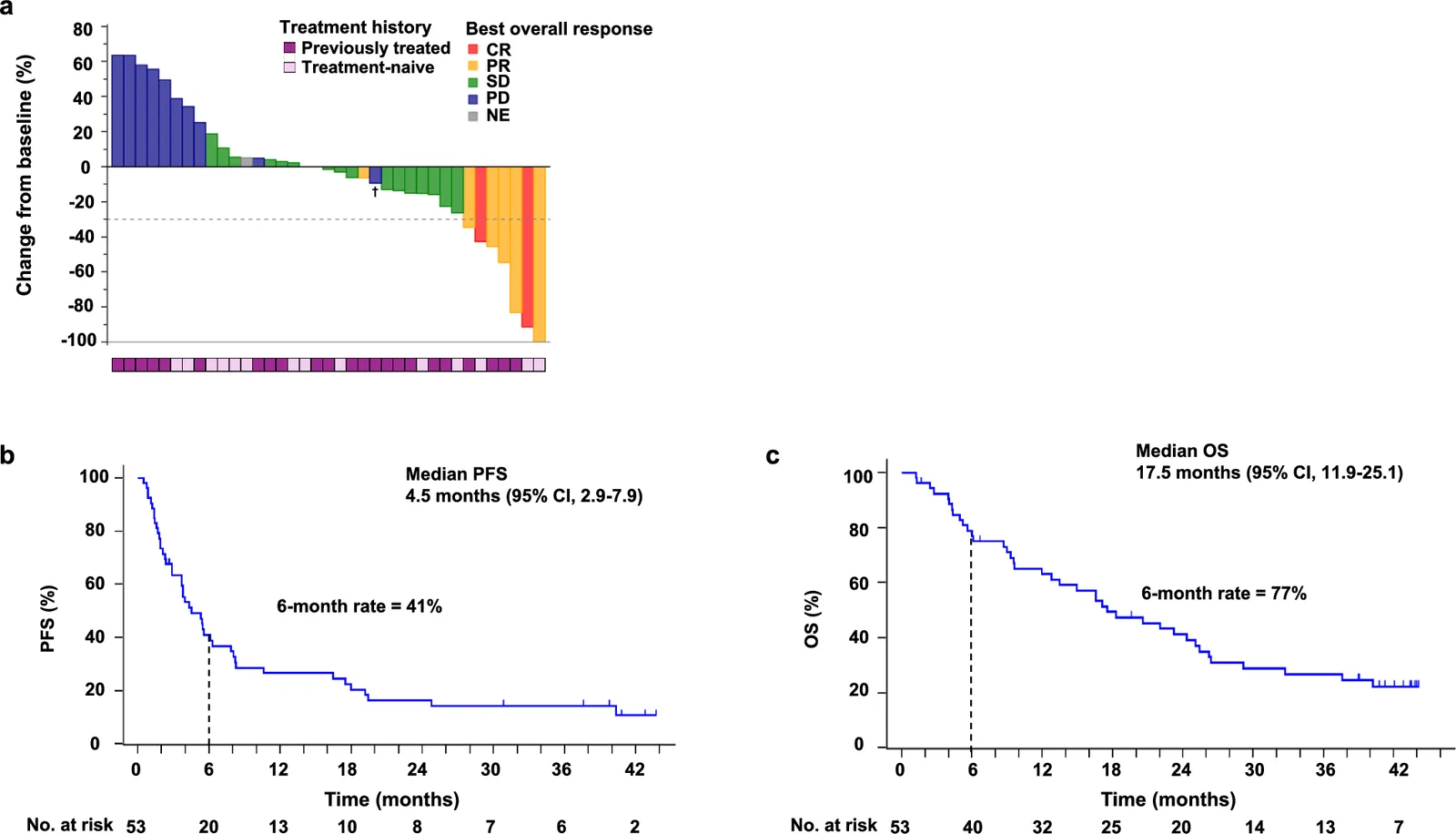
Clinical efficacy of nivolumab treatment in patients with CUP. **a** Best percentage change from baseline in the sum of the longest diameters of target lesions for the response-evaluable subset with at least one measurable lesion (n = 44); the objective response rate reported in the main text refers to the full treated population (n = 53). ^**†**^One patient with target-lesion shrinkage was classified as having progressive disease because of a new lesion. Patients are color-coded according to best overall response: CR, complete response; PR, partial response; SD, stable disease; PD, progressive disease; NE, not evaluated. **b** and **c** Kaplan–Meier curves for the probability of PFS and OS, respectively, in the overall population. Six-month survival rates are also indicated. CI confidence interval, CUP cancer of unknown primary, OS overall survival, PFS progression-free survival

At the data cutoff for this efficacy analysis (31 January 2025), the median follow-up duration was 17.0 months (range, 1.2–44.1 months). Among all patients (n = 53), 2 (3.8%) individuals had a complete response and 7 (13.2%) had a partial response, yielding an ORR of 17.0% (95% CI 8.1–29.8%) according to RECIST version 1.1 by investigator assessment (Table 2, Fig. 1a). Median PFS was 4.5 months (95% CI 2.9–7.9), with an estimated 6-month PFS rate of 41%, and median OS was 17.5 months (95% CI 11.9–25.1), with an estimated 6-month OS rate of 77% (Fig. 1b, c). Median DoR among the nine patients with an objective response was 15.0 months (95% CI 1.6 months—not reached). According to treatment history, the ORR was similar in previously treated (n = 31) and treatment-naïve (n = 22) patients (16.1% [95% CI 5.5–33.7%] vs. 18.2% [95% CI 5.2–40.3%]), whereas median PFS and median OS were 3.7 months (95% CI 1.9–5.4) and 16.6 months (95% CI 9.3–25.4) in the previously treated cohort, compared with 7.9 months (95% CI 3.9–18.0) and 22.6 months (95% CI 6.0–32.7) in the treatment-naïve cohort (Table 2). In the response-evaluable subset with at least one measurable lesion according to RECIST (n = 44), ORR was 20.5% (95% CI 9.8–35.3%), and median PFS and median OS were 4.3 months (95% CI 2.3–6.1) and 18.3 months (95% CI 9.5–25.1), respectively (Supplementary Figure S2). Response rates and survival outcomes according to treatment history were similar in this subset (Supplementary Table S3, Supplementary Figure S3).

**Table 2:** Efficacy according to treatment history

	All (n = 53)	Previously treated (n = 31)	Treatment-naïve (n = 22)
Objective response rate, n (%)	9 (17.0)	5 (16.1)	4 (18.2)
95% CI for %	8.1–29.8	5.5–33.7	5.2–40.3
Best overall response, n (%)			
Complete response	2 (3.8)	0 (0.0)	2 (9.1)
Partial response	7 (13.2)	5 (16.1)	2 (9.1)
Stable disease	18 (34.0)	10 (32.3)	8 (36.4)
Progressive disease	12 (22.6)	10 (32.3)	2 (9.1)
Non-CR/non-PD	6 (11.3)	2 (6.5)	4 (18.2)
Not evaluated	8 (15.1)	4 (12.9)	4 (18.2)
Disease control rate, n (%)	27 (50.9)	15 (48.4)	12 (54.5)
95% CI for %	36.8–64.9	30.2–66.9	32.2–75.6
Median PFS (95% CI), months	4.5 (2.9–7.9)	3.7 (1.9–5.4)	7.9 (3.9–18.0)
Median OS (95% CI), months	17.5 (11.9–25.1)	16.6 (9.3–25.4)	22.6 (6.0–32.7)

CI, confidence interval; CR, complete response; OS, overall survival; PD, progressive disease; PFS, progression-free survival; RECIST, Response Evaluation Criteria in Solid Tumors

### Treatment outcome according to baseline tumor characteristics

**Fig. 2:**
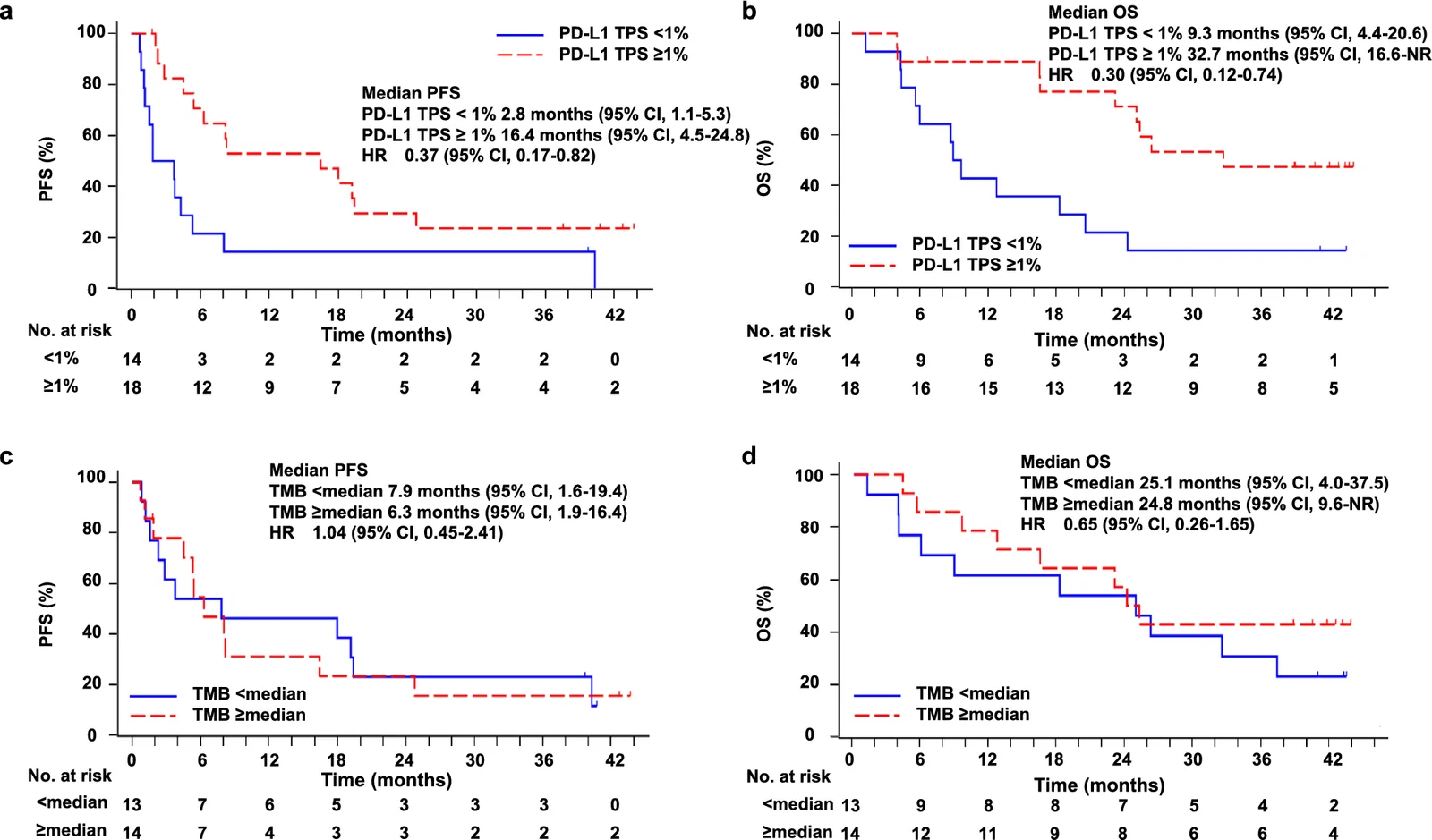
Clinical efficacy of nivolumab treatment according to PD-L1 expression or TMB in the biomarker-evaluable population. **a** and **b** Kaplan–Meier curves for the probability of PFS and OS, respectively, for patients with CUP classified according to a cutoff for PD-L1 TPS of 1% (PD-L1-evaluable patients, n = 32). **c** and **d** Kaplan–Meier curves for the probability of PFS and OS, respectively, for patients with CUP classified according to TMB (≥ vs. < the cohort median of 7.51 mutations per megabase; TMB-evaluable patients, n = 27). Hazard ratios are unadjusted and descriptive given the small subgroups. CI confidence interval, CUP cancer of unknown primary, HR hazard ratio, NR not reached, OS overall survival, PD-L1 programmed cell death–ligand 1, PFS progression-free survival, TMB tumor mutation burden, TPS tumor proportion score

We next analyzed treatment efficacy according to specific biomarkers, including PD-L1 expression, TMB, and MSI status. PD-L1 expression was evaluated in 32 patients with TPS and CPS cutoffs of 1%, 10%, and 50%. Regardless of the scoring system (TPS or CPS) or the specific cutoff level, higher PD-L1 expression was consistently associated with a higher ORR as well as a trend toward longer PFS and OS. For example, with a TPS cutoff of 1%, patients with high expression (TPS ≥ 1%) had a higher ORR (33.3% vs. 7.1%), better median PFS (16.4 months [95% CI 4.5–24.8] vs. 2.8 months [95% CI 1.1–5.3]), and longer median OS (32.7 months [95% CI 16.6—not reached] vs. 9.3 months [95% CI 4.4–20.6]) compared with those with low expression (Fig. 2a, b; Supplementary Table S4). Data for the similar favorable trends across all other evaluated cutoffs are presented in Supplementary Tables S4 to S7 and Supplementary Figures S4 to S7.

TMB was evaluated in 27 patients with available genomic data. With the median TMB value of 7.51 mutations per megabase as the cutoff, individuals were stratified into a TMB ≥ median group and a TMB < median group. This median-based dichotomization was exploratory and differs from commonly used tumor-agnostic clinical thresholds such as 10 mutations per megabase; the observed difference in ORR should therefore be interpreted with caution. The TMB ≥ median group showed a higher ORR compared with the TMB < median group (28.6% [95% CI 8.4–58.1%] vs. 7.7% [95% CI 0.2–36.0%]) (Supplementary Table S8). However, no substantial differences in survival outcomes were apparent between the two TMB groups, with median PFS values of 6.3 months (95% CI 1.9–16.4) versus 7.9 months (95% CI 1.6–19.4) and median OS values of 24.8 months (95% CI 9.6 months—not reached) versus 25.1 months (95% CI 4.0–37.5) in the TMB ≥ median and TMB < median groups, respectively (Fig. 2c, d). MSI status was evaluated in 25 patients with available tumor samples, but no MSI-high tumors were identified among these tested samples.

The potential impact of metastatic pattern on clinical outcomes was also evaluated. Among the study population, 12 individuals (22.6%) showed a metastatic pattern in which lesions were limited to multiple lymph nodes. Patients with only lymph node involvement showed better efficacy compared with those without this pattern, with an ORR of 25.0% (95% CI 5.5–57.2%) versus 14.6% (95% CI 5.6–29.2%), median PFS of 11.4 months (95% CI 2.3 months—not reached) versus 3.9 months (95% CI 2.1–6.1), and median OS of 26.4 months (95% CI 13.5 months—not reached) versus 14.9 months (95% CI 8.9–20.6), respectively (Supplementary Figure S8, Supplementary Table S9).

### Genomic landscape and predicted primary sites

To characterize the molecular background of the cohort, we performed genomic profiling for both tumor tissue (n = 28) and circulating tumor DNA (ctDNA, n = 47). In the tissue analysis, mutations in 115 unique genes were identified, with *KMT2D* (32.1%), *TP53* (32.1%), and *PIK3CA* (21.4%) being the most frequently mutated genes. The ctDNA analysis identified mutations in 32 unique genes, with *TP53* (42.6%) being the most frequently mutated gene, followed by *BRCA2* (17.0%), *APC* (14.9%), *KIT* (14.9%), and *PIK3CA* (14.9%). Recurrently mutated genes (defined as those identified in at least two patients) for both tissue and ctDNA are visualized in Supplementary Figure S9, and a complete list of all identified mutations, including those detected in only a single patient, is provided in Supplementary Table S10. The presence of these genetic alterations was not associated with the clinical benefit of nivolumab in our study cohort (data not shown).

We next performed a tissue-of-origin analysis based on gene expression and gene alteration data obtained by next-generation sequencing, which predicted a tissue of origin for 28 patients across 11 different sites (Supplementary Table S11). The most common predicted origins were non-small cell lung cancer (28.6%) and renal cell carcinoma (21.4%). We further evaluated treatment efficacy according to the approval status of ICI monotherapy by the Pharmaceuticals and Medical Devices Agency of Japan, U.S. Food and Drug Administration, and European Medicines Agency for the predicted primary sites. Patients were categorized into two groups: those predicted to have primary sites for which ICI monotherapy is approved (ICI-approved sites, including non-small cell lung cancer, renal cell carcinoma, head and neck squamous cell carcinoma, hepatocellular carcinoma, cervical cancer, and urothelial cancer; n = 21) and those with sites for which it is not approved (nonapproved sites; n = 7). No significant difference in treatment efficacy was observed between these two groups, with an ORR of 19.0% (95% CI 5.4–41.9%) in the ICI-approved sites group compared with 42.9% (95% CI 9.9–81.6%) in the nonapproved sites group. Similarly, survival outcomes were similar between the two groups, with a median PFS of 6.3 months (95% CI 2.3–18.0) versus 8.3 months (95% CI 1.9 months—not reached) and a median OS of not reached (95% CI 6.0 months—not reached) versus 24.3 months (95% CI 8.9 months—not reached) for the approved-sites and nonapproved-sites groups, respectively (Supplementary Figure S10, Supplementary Table S12).

### Exploratory immune profiling

**Fig. 3:**
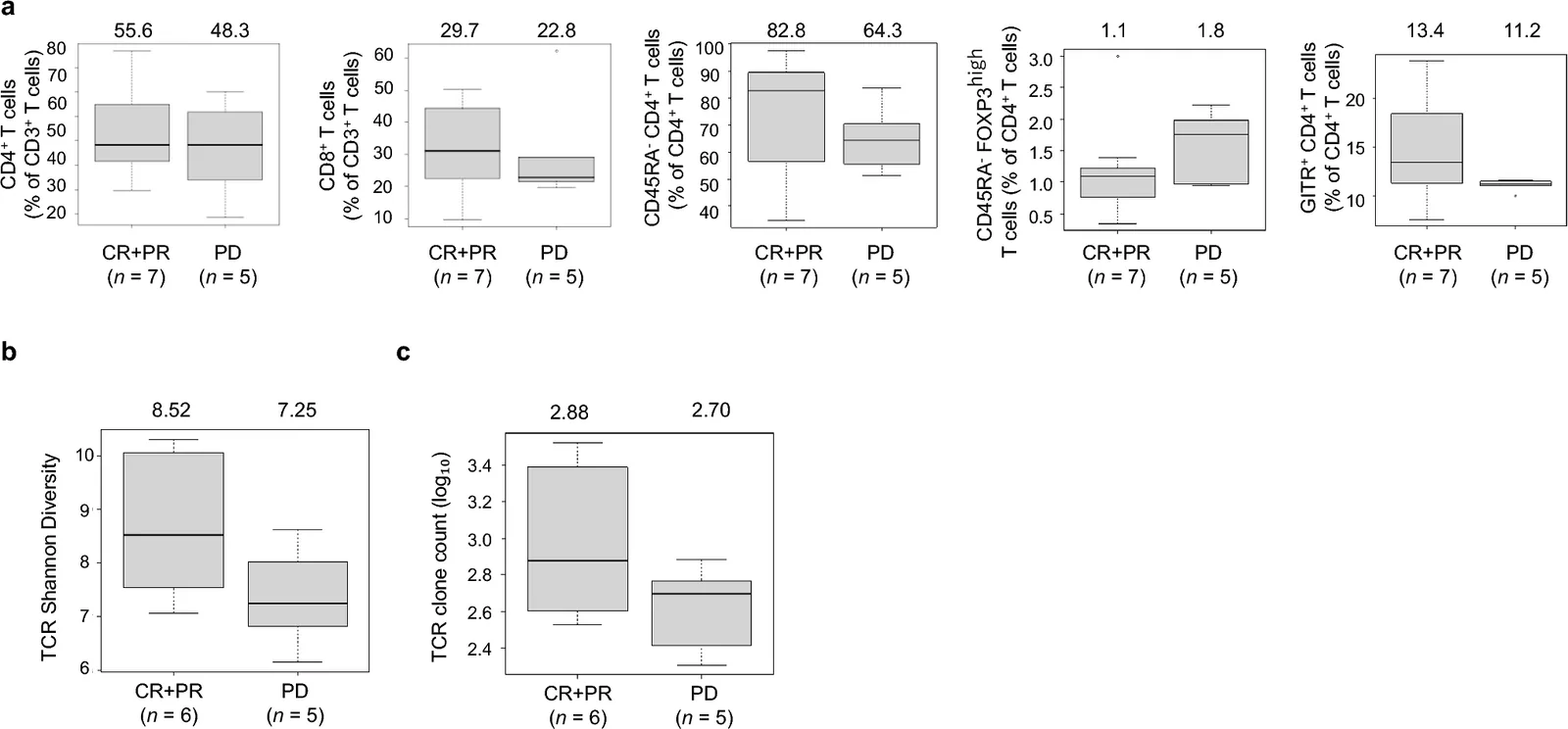
Comparison of PBMC subsets and TCR repertoire metrics in tumor tissue between responders and patients with progressive disease. **a** Flow cytometric quantitation of T-cell subsets in PBMCs isolated before nivolumab treatment from responders (patients with a complete response or partial response) and patients with progressive disease. Patients with stable disease were not included in this comparison. The horizontal line within each box represents the median, the top and bottom of the box indicate the interquartile range, and the whiskers represent the range. **b** and **c** TCR Shannon diversity (Shannon entropy) and TCR clone count (number of unique productive TCR beta clonotypes, shown on a log_10_ scale), respectively, as determined by TCR beta gene sequencing in tumor tissue obtained from responders and patients with progressive disease before nivolumab treatment. Higher values indicate a greater diversity of the TCR repertoire and a greater number of distinct clonotypes, respectively. CR complete response, PBMC peripheral blood mononuclear cell, PD progressive disease, PR partial response, TCR T-cell receptor. No formal statistical testing was performed for these comparisons, and the values shown are descriptive

Finally, we performed exploratory immune profiling with PBMCs and tumor tissue. Among a subset of 29 patients with PBMCs available before nivolumab treatment, responders (complete or partial response, n = 7) showed a higher frequency of CD45RA−CD4+ T cells and GITR+CD4+ T cells, whereas individuals with disease progression (n = 5) showed a higher frequency of CD45RA−FOXP3^high^ T cells (Fig. 3a). We also analyzed the TCR Shannon diversity and TCR clone count of T cells in both tumor tissue (n = 28) and blood samples (n = 46) obtained before treatment by TCR beta gene sequencing. For tumor tissue samples (Fig. 3b, c), the responder group (n = 6) showed numerically higher TCR Shannon diversity (median, 8.52 [95% CI 7.07–10.31] vs. 7.25 [95% CI 5.10–11.57]) and TCR clone count (log_10_; median, 2.88 [95% CI 2.53–3.53] vs. 2.70 [95% CI 2.00–3.75]) compared with the disease progression group (n = 5). In contrast to these findings for tumor tissue, pretreatment peripheral TCR Shannon diversity and TCR clone count were similar between responders (n = 9) and the disease progression group (n = 9) (Supplementary Figure S11). These data indicate that, although peripheral immune monitoring captures certain aspects of the response, the localized TCR repertoire within the tumor provides a more comprehensive view of the active immune microenvironment. Throughout these analyses, patients with stable disease were excluded from the comparisons between responders and patients with progressive disease.

## Discussion

This investigator-initiated EAP has demonstrated that nivolumab monotherapy has a manageable safety profile and provides durable clinical benefits in individuals with CUP. The ORR of 17.0% in the entire population and the corresponding rate of 20.5% in the response-evaluable subset with measurable lesions per RECIST closely mirror the results previously obtained in the pivotal investigator-initiated NivoCUP phase II trial [11]. Although the clinical utility of PD-1 blockade has been established for MSI-high or TMB-high tumors across organ types [14, 15], evidence has remained limited for patients with CUP lacking these features. Reflecting this paucity of data, current clinical practice guidelines include varying levels of endorsement. For instance, the Japanese guideline for CUP recommends nivolumab [16], whereas the ESMO guideline suggests consideration of ICIs [6], primarily as a result of reliance on a single phase II trial. Given the absence of other prospective confirmatory data, such discrepancy in recommendations is understandable. In this context, our prospective EAP provides additional prospective evidence that is descriptively consistent with the pivotal trial. Because this was an expanded access program with safety as its primary objective and no prespecified efficacy hypothesis, these findings support the reproducibility of the initial results in an independent, expanded-access cohort rather than constituting confirmatory validation.

With regard to candidate biomarkers, our exploratory analysis showed an association between higher PD-L1 expression and improved outcomes among nivolumab-treated patients. Consistent with previous data [11], patients with higher PD-L1 expression, whether evaluated by TPS or CPS, achieved better outcomes relative to those with lower expression. This reproducibility across cohorts suggests that PD-L1 expression warrants further evaluation as a candidate biomarker for CUP; however, because the present study was single-arm and lacked a control group, these data cannot distinguish a predictive from a prognostic effect. These consistent efficacy data provide preliminary support for further evaluation of a 1% cutoff. Patients with PD-L1 expression of ≥ 1% achieved an ORR of 33.3% (TPS) or 26.1% (CPS), values similar to the 30.9% and 27.9%, respectively, reported in the NivoCUP trial [11]. Importantly, these response rates in the PD-L1-positive population are similar to the historical range of 20% to 40% for platinum-doublet chemotherapy [3, 4, 17–19]. Because these observations derive from small subgroups and from cross-trial comparison with historical chemotherapy data, the possibility that a 1% threshold could inform the choice between nivolumab and platinum-based chemotherapy for PD-L1-negative patients remains a hypothesis requiring prospective validation.

The present study provides a prospective integration of anti-PD-1 efficacy for CUP with multicompartment immunologic profiling. Although the sample size was limited, exploratory analysis suggested a potential divergence between systemic and local immune repertoires. Whereas PBMC-based profiling showed certain immunologic shifts, a more notable signal was observed for intratumoral TCR clone count regarding its association with treatment response. These preliminary findings suggest the biological relevance of adaptive immunity within the tumor microenvironment of CUP, a phenomenon previously established across a wide range of solid tumors [20, 21]. Of note, the efficacy of nivolumab appeared similar regardless of whether the predicted primary site fell within an ICI-approved category; however, these subgroups were small (n = 21 vs. 7), the comparison was not statistically powered, and ICI approval status itself depends on jurisdiction, line of therapy, histology, and other biomarkers. These exploratory, hypothesis-generating observations raise the possibility that the immunologic landscape of the tumor, rather than its predicted tissue of origin, may influence ICI sensitivity in CUP, but this requires confirmation in larger studies.

The safety profile of nivolumab in the present study was consistent with its well-established tolerability across other indications. No new safety signals were identified. Given that patients with rare cancers often have limited lines of therapy, the favorable toxicity profile observed here is clinically relevant, supporting the feasibility of this treatment even in a potentially frailer real-world population. These safety observations must nevertheless be interpreted in the context of a limited observation period. Adverse events were collected only during the EAP and for 28 days after the last dose, no safety data were collected during the subsequent observational study, and the median duration of nivolumab exposure was 70 days. The absence of adverse events leading to treatment discontinuation should therefore be interpreted in the light of this restricted window of observation rather than as evidence of unusually favorable tolerability.

Several limitations of our study should be acknowledged. First, the single-arm design and the relatively small sample size, although inherent to investigations of rare diseases, limit the statistical power for subgroup analysis. Although exploratory analysis suggested interesting signals, the limited sample size precluded definitive conclusions. Second, minor differences in baseline patient characteristics between this EAP and the previous pivotal trial are apparent, reflecting real-world clinical practice. Nevertheless, the consistency of our present data with the previous results strengthens the validity of our conclusions. Third, because some patients continued nivolumab in routine clinical practice after termination of the EAP and subsequent therapies were not controlled, overall survival may have been influenced by post-protocol treatment and should be interpreted accordingly. Fourth, the more favorable outcomes observed in patients with lymph node-only disease may reflect a lower tumor burden or other favorable clinical features rather than greater nivolumab sensitivity, and should be regarded as exploratory. Fifth, all biomarker subgroup analyses were exploratory, were based on small numbers of evaluable patients, and were not adjusted for potential confounders or for multiple comparisons; in a single-arm study they therefore cannot distinguish predictive from prognostic associations. Finally, the exploratory immune profiling compared only the two extremes of response, given that patients with stable disease—a heterogeneous category under immune checkpoint inhibition—were not included; these comparisons are thus susceptible to selection bias.

In conclusion, this investigator-initiated EAP supports the reproducibility of the efficacy and safety of nivolumab for patients with CUP in an expanded-access setting. The reproducible clinical benefit, together with an exploratory association between higher PD-L1 expression and improved outcomes, represents an incremental step forward. Our data support anti-PD-1 therapy as a treatment option for individuals with this rare malignancy; internationally, however, practice remains heterogeneous and increasingly incorporates molecular profiling, and further investigation, including combination strategies, is warranted.

## Supplementary Information

Below is the link to the electronic supplementary material.

Supplementary file1 (DOCX 1387 kb)

## Data Availability

The datasets generated and/or analyzed during the current study are not publicly available because of restrictions related to participant privacy and the terms of informed consent, but they are available from the corresponding author on reasonable request.
