# Supplementary material for: Efficacy and biomarker analysis for nivolumab in cancer of unknown primary: results from an investigator-initiated expanded access program
Source: Int J Clin Oncol. 2026 Sep 24;31(11):2740–50. doi: 10.1007/s10147-026-03187-9 (PMC13710682; doi:10.1007/s10147-026-03187-9)
Supplement: Supplementary file 1 [file 10147_2026_3187_MOESM1_ESM.docx]

**Supplementary Information**

**Supplementary Methods**

**Supplementary References**

**Supplementary Figures S1 to S11**

**Supplementary Tables S1 to S12**

**Supplementary Methods**

***Immunohistochemistry***

Sections of formalin-fixed, paraffin-embedded (FFPE) tumor tissue from the study patients were subjected to immunohistochemistry with a monoclonal antibody to PD-L1 (Dako PD-L1 IHC 28-8 pharmDx test) by SRL Medisearch (Tokyo, Japan). The stained slides were evaluated independently by two board-certified pathologists who were blinded to clinical outcome. The percentage of tumor cells positive for PD-L1 was determined as the PD-L1 tumor proportion score (TPS). The combined positive score (CPS) for PD-L1 expression was also calculated as the number of PD-L1–positive cells (tumor cells, lymphocytes, macrophages) divided by the total number of tumor cells and multiplied by 100. PD-L1 positivity was defined as membranous staining at any intensity, as previously described (1). For slides with discrepant scores, the final score was determined after review of the slides and discussion by the two pathologists.

***Microsatellite instability***

Microsatellite instability (MSI) testing with an assessment method based on the quasi-monomorphic variation range (QMVR) was performed as previously described (2, 3). MSI status was classified as MSI-high on the basis of the presence of two or more unstable markers, as MSI-low on the basis of only one unstable marker, and as microsatellite stable with no unstable markers (4).

***Isolation of PBMC RNA***

RNA was extracted from peripheral blood mononuclear cells (PBMCs, purified buffy coat cells) with the use of an RNeasy Plus Mini Kit (Qiagen). RNA quantity and quality were measured with a RiboGreen RNA Assay Kit (Thermo Fisher Scientific). Extracted RNA was stored at −80°C until analysis.

***Isolation of tumor tissue DNA and RNA***

Macrodissection of FFPE tissue samples was performed to select tumor regions identified and marked by a pathologist after paraffin removal. DNA and RNA were extracted and purified with an AllPrep DNA/RNA FFPE Kit (Qiagen). DNA and RNA quantity and quality were measured with a PicoGreen dsDNA Assay Kit and a RiboGreen RNA Assay Kit (Thermo Fisher Scientific). Extracted DNA and RNA were stored at −80°C until analysis.

***DNA sequencing for tumor tissue***

Tumor-derived DNA samples were analyzed with an Oncomine Tumor Mutation Assay (Thermo Fisher Scientific) as previously described (5, 6). In brief, for library preparation, DNA was subjected to multiplex polymerase chain reaction (PCR) amplification with a Tumor Mutation Load Assay (Thermo Fisher Scientific). PCR products were ligated to Ion Xpress Barcode Adapters (Thermo Fisher Scientific) and purified with the use of Agencourt AMPure XP beads (Beckman Coulter). Purified libraries were pooled and sequenced on the Ion S5 system with the Ion 550 Chip (Thermo Fisher Scientific). Sequencing data were processed with Torrent Suite v5.12 (Thermo Fisher Scientific). Reads were aligned to the human reference genome (hg19), and variants were analyzed with Ion Reporter v5.18 (Thermo Fisher Scientific). Raw variant calls with a quality score of <25 were filtered out, and the remaining variants were manually reviewed with the Integrative Genomics Viewer (IGV, Broad Institute). Tumor mutation burden (TMB, mutations/megabase) was calculated with the Oncomine Tumor Mutation Load v2.0 workflow in Ion Reporter v5.18. Germline variants were excluded with the use of the Human Genetic Variation Database and the Exome Aggregation Consortium database.

***RNA sequencing for tumor tissue***

Tumor-derived RNA samples were analyzed with a customized targeted next-generation sequencing (NGS) panel designed with Ion AmpliSeq Designer (Thermo Fisher Scientific) as previously described (1, 5). In brief, RNA libraries were prepared from 10 ng of total RNA with the use of an Ion AmpliSeq RNA Library Kit (Thermo Fisher Scientific). PCR products were ligated to Ion Xpress Barcode Adapters (Thermo Fisher Scientific) and purified with the use of Agencourt AMPure XP beads (Beckman Coulter). Purified libraries were pooled and sequenced on the Ion S5 system with the Ion 550 Chip (Thermo Fisher Scientific). Relative gene expression levels were calculated as the ratio of raw read counts for each gene to the total read counts per sample for normalization.

***Prediction of primary site***

Prediction of the tumor primary site was performed according to a method described previously (1).

***TCR beta gene sequencing***

TCR beta gene sequencing was performed with an Oncomine TCR Beta-SR Assay (Thermo Fisher Scientific). Complementary DNA (cDNA) was synthesized from 100 ng of PBMC RNA or 200 ng of RNA from FFPE tumor tissue with the use of an Ion Torrent NGS Reverse Transcription Kit (Thermo Fisher Scientific). The cDNA was amplified with the Oncomine TCR Beta-SR Assay targeting Framework 3 and joining genes to cover the CDR3 region (average amplicon length of 80 bp). PCR products were ligated with the use of an Ion Torrent Dual Barcode Kit (Thermo Fisher Scientific) and purified with Agencourt AMPure XP beads (Beckman Coulter). Purified libraries were pooled and sequenced on the Ion S5 system with the Ion 550 Chip (Thermo Fisher Scientific). Sequencing data were processed with Torrent Suite v5.12, and TCR repertoire analysis was performed with the Oncomine TCR Beta-SR v1.4 RNA workflow in Ion Reporter v5.18 using the IMGT® database for variable (V), diversity (D), and joining (J) genes as a reference. Clone count was defined as the number of unique productive TCRβ clonotypes identified in each sample. TCR repertoire diversity was assessed using Clone Shannon Diversity (Shannon entropy), which is calculated from the relative frequencies of clonotypes. Higher Shannon Diversity values indicate a broader and more evenly distributed T-cell repertoire. In Figure 3c and Supplementary Figure S11, the TCR clone count is presented on a log₁₀ scale. Responders were defined as patients with a complete or partial response and the progressive disease group as patients whose best overall response was progressive disease; patients with stable disease were not included in these comparisons. These comparisons were descriptive; no formal statistical testing was performed and no P values were calculated. Samples that were not evaluable were excluded from the analyses, and no imputation was applied.

***DNA sequencing for plasma samples***

Cell-free DNA (cfDNA) was extracted from 2-mL plasma samples with the use of a cobas cfDNA Sample Preparation Kit (Roche Diagnostics) and was sequenced with an AVENIO cfDNA Surveillance Kit (Roche Diagnostics). Purified libraries were pooled and sequenced on an Illumina NextSeq 500 system (Illumina) with a 300-cycle high-output kit. Variants were called with AVENIO Oncology Analysis Software (version 2.0, Roche Diagnostics). All variants were manually inspected, and gene variants present at a frequency of >0.1% in population databases (Exome Aggregation Consortium, dbSNP, and 1000 Genomes) were excluded as germline variants.

***Flow cytometry***

PBMCs were washed with phosphate-buffered saline containing 2% fetal bovine serum and stained with antibodies to surface markers: CD3 (UCHT1), CD8a (RPA-T8), CD106 (STA), PD-1 (EH12.2H7), ICOS (C398.4A), and GITR (108-17) from BioLegend; and CD4 (SK3), CD45RA (HI100), and TIGIT (741182) from BD Biosciences.Viable cells were identified using eBioscience™ Fixable Viability Dye eFluor™ 780 (Thermo Fisher Scientific). Intracellular staining was performed with CTLA-4 (BNI3; BD Biosciences) and FoxP3 (259D/C7; BD Biosciences) using the FOXP3/Transcription Factor Staining Buffer Set (Thermo Fisher Scientific). Intracellular staining was performed with specific antibodies and the FOXP3/Transcription Factor Staining Buffer Set (Thermo Fisher Scientific). The samples were analyzed with a BD FACSFortessa instrument (BD Biosciences) and FlowJo software (BD Biosciences) (7).

**Supplementary References**

1.	Tanizaki J, Yonemori K, Akiyoshi K, Minami H, Ueda H, Takiguchi Y, et al. Open-label phase II study of the efficacy of nivolumab for cancer of unknown primary. Ann Oncol. 2022;33(2):216-26.

2.	Akagi K, Oki E, Taniguchi H, Nakatani K, Aoki D, Kuwata T, et al. Real-world data on microsatellite instability status in various unresectable or metastatic solid tumors. Cancer Sci. 2021;112(3):1105-13.

3.	Bando H, Okamoto W, Fukui T, Yamanaka T, Akagi K, Yoshino T. Utility of the quasi-monomorphic variation range in unresectable metastatic colorectal cancer patients. Cancer Sci. 2018;109(11):3411-5.

4.	Boland CR, Thibodeau SN, Hamilton SR, Sidransky D, Eshleman JR, Burt RW, et al. A National Cancer Institute Workshop on Microsatellite Instability for cancer detection and familial predisposition: development of international criteria for the determination of microsatellite instability in colorectal cancer. Cancer Res. 1998;58(22):5248-57.

5.	Hayashi H, Takiguchi Y, Minami H, Akiyoshi K, Segawa Y, Ueda H, et al. Site-Specific and Targeted Therapy Based on Molecular Profiling by Next-Generation Sequencing for Cancer of Unknown Primary Site: A Nonrandomized Phase 2 Clinical Trial. JAMA Oncol. 2020;6(12):1931-8.

6.	Haratani K, Nakamura A, Mamesaya N, Sawa K, Shiraishi Y, Saito R, et al. Association of immune-related adverse events with durvalumab efficacy after chemoradiotherapy in patients with unresectable Stage III non-small cell lung cancer. Br J Cancer. 2024;130(11):1783-94.

7.	Ikeda H, Kawase K, Nishi T, Watanabe T, Takenaga K, Inozume T, et al. Immune evasion through mitochondrial transfer in the tumour microenvironment. Nature. 2025;638(8049):225-36.

**Supplementary Figure S1. Consort diagram.** The diagram shows the disposition of the patients through the expanded access program (NivoCUP2, WJOG14620M) and the subsequent observational study (NivoCUP2F, WJOG14620MFS).

PD, progressive disease; RECIST, Response Evaluation Criteria in Solid Tumors.

**Supplementary Figure S2. Clinical efficacy of nivolumab in the response-evaluable subset with at least one measurable lesion according to RECIST.** Kaplan-Meier curves for probability of PFS (a), OS (b), and DoR (c) are shown.

CI, confidence interval; DoR, duration of response; NR, not reached; OS, overall survival; PFS, progression-free survival; RECIST, Response Evaluation Criteria in Solid Tumors.

**Supplementary Figure S3. Clinical efficacy of nivolumab according to prior systemic therapy status.** Kaplan-Meier curves for the probability of PFS (a) and OS (b) in the overall population as well as of PFS (c) and OS (d) in the response-evaluable subset are shown. All survival outcomes were determined by investigator assessment according to RECIST version 1.1.

CI, confidence interval; HR, hazard ratio; OS, overall survival; PFS, progression-free survival; RECIST, Response Evaluation Criteria in Solid Tumors.

**Supplementary Figure S4. Kaplan-Meier curves for probability of PFS (a, b) and OS (c, d) for patients with CUP classified according to cutoffs for PD-L1 TPS of 10% (a, c) or 50% (b, d).**

CI, confidence interval; CUP, cancer of unknown primary; HR, hazard ratio; NR, not reached; OS, overall survival; PD-L1, programmed cell death–ligand 1; PFS, progression-free survival; TPS, tumor proportion score.

**Supplementary Figure S5. Kaplan-Meier curves for probability of PFS (a–c) and OS (d–f) for patients with CUP classified according to cutoffs for PD-L1 CPS of 1% (a, d), 10% (b, e), or 50% (c, f).**

CI, confidence interval; CPS, combined positive score; CUP, cancer of unknown primary; HR, hazard ratio; NR, not reached; OS, overall survival; PD-L1, programmed cell death–ligand 1; PFS, progression-free survival.

**Supplementary Figure S6. Kaplan-Meier curves for probability of PFS (a–c) and OS (d–f) in the response-evaluable subset classified according to cutoffs for PD-L1 TPS of 1% (a, d), 10% (b, e), or 50% (c, f).**

CI, confidence interval; HR, hazard ratio; NR, not reached; OS, overall survival; PD-L1, programmed cell death–ligand 1; PFS, progression-free survival; TPS, tumor proportion score.

**Supplementary Figure S7. Kaplan-Meier curves for probability of PFS (a–c) and OS (d–f) in the response-evaluable subset classified according to cutoffs for PD-L1 CPS of 1% (a, d), 10% (b, e), or 50% (c, f).**

CI, confidence interval; CPS, combined positive score; HR, hazard ratio; NR, not reached; OS, overall survival; PD-L1, programmed cell death–ligand 1; PFS, progression-free survival.

**Supplementary Figure S8. Kaplan-Meier curves for probability of PFS (a) and OS (b) for patients classified according to metastatic pattern.**

CI, confidence interval; HR, hazard ratio; NR, not reached; OS, overall survival; PFS, progression-free survival.

**Supplementary Figure S9. Frequency of mutations detected in tumor tissue and blood samples.**

**Supplementary Figure S10. Kaplan-Meier curves for probability of PFS (a) and OS (b) for patients classified according to the approval status of ICI monotherapy for the predicted primary sites.**

CI, confidence interval; HR, hazard ratio; ICI, immune checkpoint inhibitor; NR, not reached; OS, overall survival; PFS, progression-free survival.

**Supplementary Figure S11. Comparison of peripheral TCR Shannon diversity (a) and TCR clone count (b) in PBMCs between responders (patients with a complete response or partial response) and patients with progressive disease.** The horizontal line within each box represents the median, the top and bottom of the box indicate the interquartile range, and the whiskers represent the range. Patients with stable disease were not included in this comparison. No formal statistical testing was performed for these comparisons, and the values shown are descriptive.

PBMC, peripheral blood mononuclear cell; TCR, T cell receptor.

**Supplementary Table S1. Baseline demographic and disease characteristics**

| Characteristic | Overall  (*n* = 53) | Previously treated  (*n* = 31) | Previously untreated  (*n* = 22) |
| --- | --- | --- | --- |
| Median age (range), years | 62.0 (24–84) | 63.5 (24–83) | 64.0 (30–84) |
| Sex |  |  |  |
| Male | 31 (58.5) | 15 (48.4) | 16 (72.7) |
| Female | 22 (41.5) | 16 (51.6) | 6 (27.3) |
| ECOG performance status |  |  |  |
| 0 | 22 (41.5) | 15 (48.4) | 7 (31.8) |
| 1 | 30 (56.6) | 15 (48.4) | 15 (68.2) |
| 2 | 1 (1.9) | 1 (3.2) | 0 |
| Histology |  |  |  |
| Well or moderately differentiated adenocarcinoma | 4 (7.5) | 2 (6.5) | 2 (9.1) |
| Poorly differentiated adenocarcinoma | 8 (15.1) | 4 (12.9) | 4 (18.2) |
| Adenocarcinoma (differentiation unknown) | 18 (34.0) | 11 (35.5) | 7 (31.8) |
| Squamous cell carcinoma | 11 (20.8) | 5 (16.1) | 6 (27.3) |
| Undifferentiated carcinoma | 2 (3.8) | 2 (3.5) | 0 (0.0) |
| Other | 10 (18.9) | 7 (2.3) | 3 (13.6) |
| Number of metastatic sites |  |  |  |
| 1 | 23 (43.4) | 11 (35.5) | 12 (54.5) |
| 2 | 18 (34.0) | 13 (41.9) | 5 (22.7) |
| ≥3 | 12 (22.6) | 7 (22.5) | 5 (22.7) |
| Lymph nodes only |  |  |  |
| Yes | 12 (22.6) | 5 (16.1) | 7 (31.8) |
| No | 41 (77.4) | 26 (83.9) | 15 (68.2) |
| Prior lines of chemotherapy |  |  |  |
| 1 | 22 (41.5) | 22 (71.0) |  |
| 2 | 5 (9.4) | 5 (16.1) |  |
| ≥3 | 4 (7.5) | 4 (12.9) |  |
| Prior radiotherapy |  |  |  |
| Yes | 15 (28.3) | 9 (29.0) | 6 (27.3) |
| No | 38 (71.7) | 22 (71.0) | 16 (72.7) |
| Metastatic sites |  |  |  |
| Liver | 13 (24.5) | 8 (25.8) | 5 (22.7) |
| Lung | 10 (18.9) | 7 (22.6) | 3 (13.6) |
| Bone | 14 (26.4) | 8 (25.8) | 6 (27.3) |
| Pleura | 3 (5.7) | 2 (6.5) | 1 (4.5) |
| Peritoneum | 14 (26.4) | 8 (25.8) | 6 (27.3) |
| Skin | 1 (1.9) | 1 (3.2) | 0 (0.0) |
| Brain | 0 (0.0) | 0 (0.0) | 0 (0.0) |
| Adrenal gland | 4 (7.5) | 2 (6.5) | 2 (9.1) |
| Lymph node | 33 (62.3) | 20 (64.5) | 13 (59.1) |
| Other | 10 (18.9) | 5 (16.1) | 5 (22.7) |

With the exception of age, all data are *n* (%).

ECOG, Eastern Cooperative Oncology Group.

**Supplementary Table S2. Prior chemotherapy regimens for previously treated patients with CUP (*n* =31)**

| Prior chemotherapy | *n* (%) |
| --- | --- |
| Platinum-based regimens |  |
| CBDCA+PTX | 17 (54.8) |
| CDDP+GEM | 5 (16.1) |
| SOX | 2 (6.5) |
| CAPOX | 1 (2.2) |
| CAPOX+BEV | 1 (2.2) |
| CBDCA+DTX | 1 (3.2) |
| CBDCA+PTX+BEV | 1 (2.2) |
| CDDP+PTX+BEV | 1 (2.2) |
| FOLFIRINOX | 1 (2.2) |
| FOLFOX | 1 (2.2) |
| FP | 1 (2.2) |
| Other |  |
| DTX | 2 (6.5) |
| GEM | 2 (6.5) |
| S-1 | 2 (6.5) |
| Capecitabine | 1 (2.2) |
| CPT-11 | 1 (2.2) |
| DOX+BEV | 1 (2.2) |
| GEM+BEV | 1 (2.2) |
| GEM+nab-PTX | 1 (2.2) |
| nal-IRI+5-FU/LV | 1 (2.2) |

Patients with more than one prior therapy are included in the listing for each of their regimens.

BEV, bevacizumab; CAPOX, oxaliplatin and capecitabine; CBDCA, carboplatin; CDDP, cisplatin; CPT-11, irinotecan; CUP, cancer of unknown primary; DOX, doxorubicin; DTX, docetaxel; FOLFIRINOX, oxaliplatin, irinotecan, infusional and bolus 5-fluorouracil, and leucovorin; FOLFOX, oxaliplatin, infusional and bolus 5-fluorouracil, and leucovorin; FP, cisplatin and 5-fluorouracil; 5-FU, 5-fluorouracil; GEM, gemcitabine; LV, folinic acid; nab-PTX, nab-paclitaxel; nal-IRI, liposomal irinotecan; PTX, paclitaxel; SOX, oxaliplatin and S-1.

**Supplementary Table S3. Efficacy according to treatment history in the response-evaluable subset**

|  | RES (n = 44) | Previously treated (n = 26) | Treatment-naïve (n = 18) |
| --- | --- | --- | --- |
| Objective response rate, n (%) | 9 (20.5) | 5 (19.2) | 4 (22.2) |
| 95% CI for % | 9.8–35.3 | 6.6–39.4 | 6.4–47.6 |
| Best overall response, n (%) |  |  |  |
| Complete response | 2 (4.5) | 0 (0.0) | 2 (11.1) |
| Partial response | 7 (15.9) | 5 (19.2) | 2 (11.1) |
| Stable disease | 18 (40.9) | 10 (38.5) | 8 (44.4) |
| Progressive disease | 10 (22.7) | 8 (30.8) | 2 (11.1) |
| Not evaluated | 7 (15.9) | 3 (11.5) | 4 (22.2) |
| Disease control rate, n (%) | 27 (61.4) | 15 (57.7) | 12 (66.7) |
| 95% CI for % | 45.5–75.6 | 36.9–76.6 | 41.0–86.7 |
| Median PFS (95% CI), months | 4.3 (2.3–6.1) | 3.7 (1.8–5.3) | 7.9 (2.9–19.1) |
| Median OS (95% CI), months | 18.3 (9.5–25.1) | 16.6 (9.3–24.3) | 24.1 (5.2–37.6) |

CI, confidence interval; CR, complete response; OS, overall survival; PD, progressive disease; PFS, progression-free survival; RECIST, Response Evaluation Criteria in Solid Tumors; RES, response-evaluable subset. The response-evaluable subset comprised patients with at least one measurable lesion according to RECIST version 1.1. Previously treated patients were defined as those who had received at least one platinum-containing chemotherapy regimen and were refractory to or intolerant of it. Analyses according to treatment history were prespecified and are descriptive and exploratory, with no formal between-group comparison and no adjustment for multiple comparisons.

**Supplementary Table S4. Best overall response for all patients with CUP according to PD-L1 TPS**

|  | PD-L1 TPS | | | | | | |
| --- | --- | --- | --- | --- | --- | --- | --- |
|  | <1% | ≥1% | | <10% | ≥10% | <50% | ≥50% |
| *n* (%) | 14 (43.8) | 18 (56.3) | | 22 (68.8) | 10 (31.3) | 26 (81.3) | 6 (18.8) |
| Objective response rate, *n* (%) | 1 (7.1) | 6 (33.3) | | 1 (4.5) | 6 (60.0) | 1 (3.8) | 6 (100.0) |
| 95% CI for % | 0.2–33.9 | 13.3–59.0 | | 0.1–22.8 | 26.2–87.8 | 0.1–19.6 | 54.1–100.0 |
| *P* value | 0.104 | | | 0.001 | | <0.001 | |
| Best overall response, *n* (%) |  |  | |  |  |  |  |
| Complete response | 0 (0.0) | 2 (11.1) | | 0 (0.0) | 2 (20.0) | 0 (0.0) | 2 (33.3) |
| Partial response | 1 (7.1) | 4 (22.2) | | 1 (4.5) | 4 (40.0) | 1 (3.8) | 4 (66.7) |
| Stable disease | 4 (28.6) | 6 (33.3) | | 8 (36.4) | 2 (20.0) | 10 (38.5) | 0 (0.0) |
| Progressive disease | 6 (42.9) | 0 (0.0) | | 6 (27.3) | 0 (0.0) | 6 (23.1) | 0 (0.0) |
| Non-CR/non-PD | 1 (7.1) | 2 (11.1) | 2 (9.1) | | 1 (10.0) | 3 (11.5) | 0 (0.0) |
| Not evaluated | 2 (14.3) | 4 (22.2) | 5 (22.7) | | 1 (10.0) | 6 (23.1) | 0 (0.0) |
| Disease control rate, *n* (%) | 5 (35.7) | 12 (66.7) | 9 (40.9%) | | 8 (80.0) | 11 (42.3) | 6 (100.0) |
| 95% CI for % | 12.8–64.9 | 41.0–86.7 | 20.7–63.6 | | 44.4–97.5 | 23.4–63.1 | 54.1–100.0 |

*P* values for comparison of objective response rate at each PD-L1 TPS cutoff were determined with Fisher’s exact test.

CI, confidence interval; CR, complete response; CUP, cancer of unknown primary; PD, progressive disease: PD-L1, programmed cell death–ligand 1; TPS, tumor proportion score.

**Supplementary Table S5. Best overall response for all patients with CUP according to PD-L1 CPS**

|  | PD-L1 CPS | | | | | | |
| --- | --- | --- | --- | --- | --- | --- | --- |
|  | <1% | ≥1% | | <10% | ≥10% | <50% | ≥50% |
| *n* (%) | 9 (28.1) | 23 (71.9) | | 20 (62.5) | 12 (37.5) | 26 (81.2) | 6 (18.8) |
| Objective response rate, *n* (%) | 1 (11.1) | 6 (26.1) | | 1 (5.0) | 6 (50.0) | 1 (3.8) | 6 (100.0) |
| 95% CI for % | 0.3–48.2 | 10.2–48.4 | | 0.1–24.9 | 21.1–78.9 | 0.1–19.6 | 54.1–100.0 |
| *P* value | 0.64 | | | 0.006 | | <0.001 | |
| Best overall response, *n* (%) |  |  | |  |  |  |  |
| Complete response | 0 (0.0) | 2 (8.7) | | 0 (0.0) | 2 (16.7) | 0 (0.0) | 2 (33.3) |
| Partial response | 1 (11.1) | 4 (17.4) | | 1 (5.0) | 4 (33.3) | 1 (3.8) | 4 (66.7) |
| Stable disease | 3 (33.3) | 7 (30.4) | | 6 (30.0) | 4 (33.3) | 10 (38.5) | 0 (0.0) |
| Progressive disease | 5 (55.6) | 1 (4.3) | | 6 (30.0) | 0 (0.0) | 6 (23.1) | 0 (0.0) |
| Non-CR/non-PD | 0 (0.0) | 3 (13.0) | 2 (10.0) | | 1 (8.3) | 3 (11.5) | 0 (0.0) |
| Not evaluated | 0 (0.0) | 6 (26.1) | 5 (25.0) | | 1 (8.3) | 6 (23.1) | 0 (0.0) |
| Disease control rate, *n* (%) | 4 (44.4) | 13 (56.5) | 7 (35.0%) | | 10 (83.3) | 11 (42.3) | 6 (100.0) |
| 95% CI for % | 13.7–78.8 | 34.5–76.8 | 15.4–59.2 | | 51.6–97.9 | 23.4–63.1 | 54.1–100.0 |

*P* values for comparison of objective response rate at each PD-L1 CPS cutoff were determined with Fisher’s exact test.

CI, confidence interval; CPS, combined positive score; CR, complete response; CUP, cancer of unknown primary; PD, progressive disease: PD-L1, programmed cell death–ligand 1.

**Supplementary Table S6. Best overall response for the response-evaluable subset according to PD-L1 TPS**

|  | PD-L1 TPS | | | | | | |
| --- | --- | --- | --- | --- | --- | --- | --- |
|  | <1% | ≥1% | | <10% | ≥10% | <50% | ≥50% |
| *n* (%) | 11 (42.3) | 15 (57.7) | | 17 (65.4) | 9 (34.6) | 20 (76.9) | 6 (23.1) |
| Objective response rate, *n* (%) | 1 (9.1) | 6 (40.0) | | 1 (5.9) | 6 (66.7) | 1 (5.0) | 6 (100.0) |
| 95% CI for % | 0.2–41.3 | 16.3–67.7 | | 0.1–28.7 | 29.9–92.5 | 0.1–24.9 | 54.1–100.0 |
| *P* value | 0.178 | | | 0.002 | | <0.001 | |
| Best overall response, *n* (%) |  |  | |  |  |  |  |
| Complete response | 0 (0.0) | 2 (13.3) | | 0 (0.0) | 2 (22.2) | 0 (0.0) | 2 (33.3) |
| Partial response | 1 (9.1) | 4 (26.7) | | 1 (5.9) | 4 (44.4) | 1 (5.0) | 4 (66.7) |
| Stable disease | 4 (36.4) | 6 (40.0) | | 8 (47.1) | 2 (22.2) | 10 (50.0) | 0 (0.0) |
| Progressive disease | 4 (36.4) | 0 (0.0) | | 4 (23.5) | 0 (0.0) | 4 (20.0) | 0 (0.0) |
| Not evaluated | 2 (18.2) | 3 (20.0) | 4 (23.5) | | 1 (11.1) | 5 (25.0) | 0 (0.0) |
| Disease control rate, *n* (%) | 5 (45.5) | 12 (80.0) | 9 (52.9%) | | 8 (88.9) | 11 (55.0) | 6 (100.0) |
| 95% CI for % | 16.7–76.6 | 51.9–95.7 | 27.8–77.0 | | 51.8–99.7 | 31.5–76.9 | 54.1–100.0 |

*P* values for comparison of objective response rate at each PD-L1 TPS cutoff were determined with Fisher’s exact test.

CI, confidence interval; PD-L1, programmed cell death–ligand 1; TPS, tumor proportion score.

**Supplementary Table S7. Best overall response for the response-evaluable subset according to PD-L1 CPS**

|  | PD-L1 CPS | | | | | | |
| --- | --- | --- | --- | --- | --- | --- | --- |
|  | <1% | ≥1% | | <10% | ≥10% | <50% | ≥50% |
| *n* (%) | 7 (26.9) | 19 (73.1) | | 15 (57.7) | 11 (42.3) | 20 (76.9) | 6 (23.1) |
| Objective response rate, *n* (%) | 1 (14.3) | 6 (31.6) | | 1 (6.7) | 6 (54.5) | 1 (5.0) | 6 (100.0) |
| 95% CI for % | 0.4–57.9 | 12.6–56.6 | | 0.2–31.9 | 23.4–83.3 | 0.1–24.9 | 54.1–100.0 |
| *P* value | 0.629 | | | 0.021 | | <0.001 | |
| Best overall response, *n* (%) |  |  | |  |  |  |  |
| Complete response | 0 (0.0) | 2 (10.5) | | 0 (0.0) | 2 (18.2) | 0 (0.0) | 2 (33.3) |
| Partial response | 1 (14.3) | 4 (21.1) | | 1 (6.7) | 4 (36.4) | 1 (5.0) | 4 (66.7) |
| Stable disease | 3 (42.9) | 7 (36.8) | | 6 (40.0) | 4 (36.4) | 10 (50.0) | 0 (0.0) |
| Progressive disease | 3 (42.9) | 1 (5.3) | | 4 (26.7) | 0 (0.0) | 4 (20.0) | 0 (0.0) |
| Not evaluated | 0 (0.0) | 5 (26.3) | 4 (26.7) | | 1 (9.1) | 5 (25.0) | 0 (0.0) |
| Disease control rate, *n* (%) | 4 (57.1) | 13 (68.4) | 7 (46.7%) | | 10 (90.9) | 11 (55.0) | 6 (100.0) |
| 95% CI for % | 18.4–90.1 | 43.4–87.4 | 21.3–73.4 | | 58.7–99.8 | 31.5–76.9 | 54.1–100.0 |

*P* values for comparison of objective response rate at each PD-L1 CPS cutoff were determined with Fisher’s exact test.

CI, confidence interval; CPS, combined positive score; PD-L1, programmed cell death–ligand 1.

**Supplementary Table S8. Best overall response for all patients with CUP and the response-evaluable subset according to TMB**

|  | All  (*n* = 27) | | RES  (*n* = 22) | |
| --- | --- | --- | --- | --- |
|  | TMB <median  (*n* = 13) | TMB ≥median  (*n* = 14) | TMB <median  (*n* = 12) | TMB ≥median  (*n* = 10) |
| Objective response rate, *n* (%) | 1 (7.7) | 4 (28.6) | 1 (8.3) | 4 (40.0) |
| 95% CI for % | 0.2–36.0 | 8.4–58.1 | 0.2–38.5 | 12.2–73.8 |
| Best overall response, *n* (%) |  |  |  |  |
| Complete response | 1 (7.7) | 1 (7.1) | 1 (8.3) | 1 (10.0) |
| Partial response | 0 (0.0) | 3 (21.4) | 0 (0.0) | 3 (30.0) |
| Stable disease | 7 (53.8) | 3 (21.4) | 7 (58.3) | 3 (30.0) |
| Progressive disease | 2 (15.4) | 3 (21.4) | 2 (16.7) | 2 (20.0) |
| Non-CR/non-PD | 1 (7.7) | 2 (14.3) |  |  |
| Not evaluated | 2 (15.4) | 2 (14.3) | 2 (16.7) | 1 (10.0) |
| Disease control rate, *n* (%) | 8 (61.5) | 7 (50.0) | 8 (66.7) | 7 (70.0) |
| 95% CI for % | 31.6–86.1 | 23.0–77.0 | 34.9–90.1 | 34.8–93.3 |
| Median PFS (95% CI), months | 7.9 (1.6–19.4) | 6.3 (1.9–16.4) | 5.8 (1.2–40.3) | 5.4 (1.1–24.8) |
| Median OS (95% CI), months | 25.1 (4.0–37.5) | 24.8 (9.6–NR) | 21.7 (4.0–NR) | NR (5.6–NR) |

CI, confidence interval; CR, complete response; CUP, cancer of unknown primary; NR, not reached; OS, overall survival; PD, progressive disease; PFS, progression-free survival; RES, response-evaluable subset; TMB, tumor mutation burden.

**Supplementary Table S9.** **Best overall response for all patients with CUP and the response-evaluable subset according to metastatic pattern**

|  | All  (*n* = 53) | | RES  (*n* = 44) | |
| --- | --- | --- | --- | --- |
|  | Multiple LN metastasis only  (*n* = 12) | Other  (*n* = 41) | Multiple LN metastasis only  (*n* = 9) | Other  (*n* = 35) |
| Objective response rate, *n* (%) | 3 (25.0) | 6 (14.6) | 3 (33.3) | 6 (17.1) |
| 95% CI for % | 5.5–57.2 | 5.6–29.2 | 7.5–70.1 | 6.6–33.6 |
| Best overall response, *n* (%) |  |  |  |  |
| Complete response | 2 (16.7) | 0 (0.0) | 2 (22.2) | 0 (0.0) |
| Partial response | 1 (8.3) | 6 (14.6) | 1 (11.1) | 6 (17.1) |
| Stable disease | 4 (33.3) | 14 (34.1) | 4 (44.4) | 14 (40.0) |
| Progressive disease | 1 (8.3) | 11 (26.8) | 1 (11.1) | 9 (25.7) |
| Non-CR/non-PD | 2 (16.7) | 4 (9.8) |  |  |
| Not evaluated | 2 (16.7) | 6 (14.6) | 1 (11.1) | 6 (17.1) |
| Disease control rate, *n* (%) | 7 (58.3) | 20 (48.8) | 7 (77.8) | 20 (57.1) |
| 95% CI for % | 27.7–84.8 | 32.9–64.9 | 40.0–97.2 | 39.4–73.7 |
| Median PFS (95% CI), months | 11.4 (2.3–NR) | 3.9 (2.1–6.1) | 4.5 (1.8–NR) | 3.8 (2.1–6.1) |
| Median OS (95% CI), months | 26.4 (13.5–NR) | 14.9 (8.9–20.6) | 40.2 (4.0–NR) | 16.5 (6.1–22.0) |

CI, confidence interval; CR, complete response; CUP, cancer of unknown primary; LN, lymph node; NR, not reached; OS, overall survival; PD, progressive disease; PFS, progression-free survival; RES, response-evaluable subset.

**Supplementary Table S10. Frequency of mutations detected in tumor tissue (*n* = 28) and blood samples (*n* = 47)**

| Gene | Tissue count | Tissue frequency (%) | Tissue mutation details | Blood count | Blood frequency (%) | Blood mutation details |
| --- | --- | --- | --- | --- | --- | --- |
| TP53 | 9 | 32.1 | missense(4), nonsense(4), fs(1) | 20 | 42.6 | SNV(20) |
| KMT2D | 9 | 32.1 | nonsense(4), nonfs(2), missense(2), fs(1) | 0 | 0 | 0 |
| PIK3CA | 6 | 21.4 | missense(6) | 7 | 14.9 | SNV(7) |
| ARID1A | 5 | 17.9 | missense(3), fs(1), nonsense(1) | 0 | 0 | 0 |
| ARID2 | 5 | 17.9 | fs(2), nonsense(2), missense(1) | 0 | 0 | 0 |
| KMT2C | 5 | 17.9 | nonsense(3), missense(2) | 0 | 0 | 0 |
| KAT6A | 4 | 14.3 | nonfs(3), missense(1) | 0 | 0 | 0 |
| ZNF521 | 3 | 10.7 | missense(3) | 6 | 12.8 | SNV(6) |
| CSMD3 | 3 | 10.7 | missense(2), nonsense(1) | 2 | 4.3 | SNV(2) |
| PMS1 | 3 | 10.7 | fs(2), missense(1) | 0 | 0 | 0 |
| FN1 | 3 | 10.7 | missense(3) | 0 | 0 | 0 |
| KAT6B | 3 | 10.7 | missense(3) | 0 | 0 | 0 |
| SYNE1 | 3 | 10.7 | missense(2), nonsense(1) | 0 | 0 | 0 |
| KIT | 2 | 7.1 | missense(2) | 7 | 14.9 | SNV(7) |
| APC | 2 | 7.1 | missense(2) | 7 | 14.9 | SNV(7) |
| KRAS | 2 | 7.1 | missense(2) | 5 | 10.6 | SNV(5) |
| NFE2L2 | 2 | 7.1 | missense(2) | 4 | 8.5 | SNV(4) |
| CDKN2A | 2 | 7.1 | nonsense(2) | 1 | 2.1 | SNV(1) |
| EP400 | 2 | 7.1 | missense(2) | 0 | 0 | 0 |
| SMARCA4 | 2 | 7.1 | missense(1), nonsense(1) | 0 | 0 | 0 |
| EPHA3 | 2 | 7.1 | missense(2) | 0 | 0 | 0 |
| NUMA1 | 2 | 7.1 | missense(2) | 0 | 0 | 0 |
| TET2 | 2 | 7.1 | missense(2) | 0 | 0 | 0 |
| PKHD1 | 2 | 7.1 | missense(2) | 0 | 0 | 0 |
| PLEKHG5 | 2 | 7.1 | missense(2) | 0 | 0 | 0 |
| RNF213 | 2 | 7.1 | missense(2) | 0 | 0 | 0 |
| AR | 2 | 7.1 | missense(2) | 0 | 0 | 0 |
| AKAP9 | 2 | 7.1 | missense(2) | 0 | 0 | 0 |
| PTPRD | 2 | 7.1 | missense(2) | 0 | 0 | 0 |
| TGM7 | 2 | 7.1 | missense(2) | 0 | 0 | 0 |
| NUP214 | 2 | 7.1 | missense(2) | 0 | 0 | 0 |
| MARK1 | 2 | 7.1 | missense(2) | 0 | 0 | 0 |
| ATM | 2 | 7.1 | missense(1), nonsense(1) | 0 | 0 | 0 |
| TAF1L | 2 | 7.1 | missense(2) | 0 | 0 | 0 |
| ABL2 | 2 | 7.1 | missense(2) | 0 | 0 | 0 |
| NLRP1 | 2 | 7.1 | missense(2) | 0 | 0 | 0 |
| BCL9 | 2 | 7.1 | nonsense(1), missense(1) | 0 | 0 | 0 |
| EP300 | 2 | 7.1 | missense(2) | 0 | 0 | 0 |
| ADAMTS20 | 2 | 7.1 | missense(2) | 0 | 0 | 0 |
| RB1 | 2 | 7.1 | missense(1), nonsense(1) | 0 | 0 | 0 |
| RECQL4 | 2 | 7.1 | missense(2) | 0 | 0 | 0 |
| KMT2A | 2 | 7.1 | missense(2) | 0 | 0 | 0 |
| PIK3CG | 1 | 3.6 | missense(1) | 5 | 10.6 | SNV(5) |
| RET | 1 | 3.6 | fs(1) | 5 | 10.6 | SNV(5) |
| ERBB2 | 1 | 3.6 | missense(1) | 5 | 10.6 | SNV(5) |
| ALK | 1 | 3.6 | missense(1) | 5 | 10.6 | SNV(5) |
| PDGFRA | 1 | 3.6 | missense(1) | 4 | 8.5 | SNV(4) |
| ROS1 | 1 | 3.6 | missense(1) | 2 | 4.3 | SNV(2) |
| FBXW7 | 1 | 3.6 | missense(1) | 2 | 4.3 | SNV(2) |
| PBX1 | 1 | 3.6 | missense(1) | 0 | 0 | 0 |
| DICER1 | 1 | 3.6 | missense(1) | 0 | 0 | 0 |
| SDHA | 1 | 3.6 | missense(1) | 0 | 0 | 0 |
| IRS2 | 1 | 3.6 | missense(1) | 0 | 0 | 0 |
| HLF | 1 | 3.6 | missense(1) | 0 | 0 | 0 |
| TAF1 | 1 | 3.6 | missense(1) | 0 | 0 | 0 |
| PIK3CD | 1 | 3.6 | missense(1) | 0 | 0 | 0 |
| BIVM-ERCC5 | 1 | 3.6 | missense(1) | 0 | 0 | 0 |
| BCL2L1 | 1 | 3.6 | missense(1) | 0 | 0 | 0 |
| AKT3 | 1 | 3.6 | missense(1) | 0 | 0 | 0 |
| MAML2 | 1 | 3.6 | missense(1) | 0 | 0 | 0 |
| CDK8 | 1 | 3.6 | missense(1) | 0 | 0 | 0 |
| CTNNA1 | 1 | 3.6 | missense(1) | 0 | 0 | 0 |
| PDGFRB | 1 | 3.6 | missense(1) | 0 | 0 | 0 |
| NSD1 | 1 | 3.6 | missense(1) | 0 | 0 | 0 |
| SUFU | 1 | 3.6 | missense(1) | 0 | 0 | 0 |
| CDK12 | 1 | 3.6 | fs(1) | 0 | 0 | 0 |
| FGFR3 | 1 | 3.6 | missense(1) | 0 | 0 | 0 |
| PML | 1 | 3.6 | missense(1) | 0 | 0 | 0 |
| TNFRSF14 | 1 | 3.6 | missense(1) | 0 | 0 | 0 |
| NFKB2 | 1 | 3.6 | missense(1) | 0 | 0 | 0 |
| ERBB3 | 1 | 3.6 | missense(1) | 0 | 0 | 0 |
| CYLD | 1 | 3.6 | missense(1) | 0 | 0 | 0 |
| CDH5 | 1 | 3.6 | missense(1) | 0 | 0 | 0 |
| PAX3 | 1 | 3.6 | missense(1) | 0 | 0 | 0 |
| SETD2 | 1 | 3.6 | missense(1) | 0 | 0 | 0 |
| KDR | 1 | 3.6 | nonsense(1) | 0 | 0 | 0 |
| ETV1 | 1 | 3.6 | missense(1) | 0 | 0 | 0 |
| FGFR2 | 1 | 3.6 | missense(1) | 0 | 0 | 0 |
| ERBB4 | 1 | 3.6 | missense(1) | 0 | 0 | 0 |
| BLM | 1 | 3.6 | missense(1) | 0 | 0 | 0 |
| ARNT | 1 | 3.6 | missense(1) | 0 | 0 | 0 |
| GNAS | 1 | 3.6 | missense(1) | 0 | 0 | 0 |
| DST | 1 | 3.6 | missense(1) | 0 | 0 | 0 |
| NOTCH1 | 1 | 3.6 | missense(1) | 0 | 0 | 0 |
| NTRK1 | 1 | 3.6 | missense(1) | 0 | 0 | 0 |
| JAK1 | 1 | 3.6 | missense(1) | 0 | 0 | 0 |
| SMAD4 | 1 | 3.6 | fs(1) | 0 | 0 | 0 |
| THBS1 | 1 | 3.6 | missense(1) | 0 | 0 | 0 |
| AFF3 | 1 | 3.6 | fs(1) | 0 | 0 | 0 |
| TBX22 | 1 | 3.6 | missense(1) | 0 | 0 | 0 |
| LTK | 1 | 3.6 | missense(1) | 0 | 0 | 0 |
| TSHR | 1 | 3.6 | missense(1) | 0 | 0 | 0 |
| GATA1 | 1 | 3.6 | missense(1) | 0 | 0 | 0 |
| HRAS | 1 | 3.6 | missense(1) | 0 | 0 | 0 |
| ADGRL3 | 1 | 3.6 | missense(1) | 0 | 0 | 0 |
| EPHB6 | 1 | 3.6 | missense(1) | 0 | 0 | 0 |
| PLAG1 | 1 | 3.6 | missense(1) | 0 | 0 | 0 |
| KDM6A | 1 | 3.6 | missense(1) | 0 | 0 | 0 |
| PTEN | 1 | 3.6 | missense(1) | 0 | 0 | 0 |
| NF1 | 1 | 3.6 | nonsense(1) | 0 | 0 | 0 |
| FLCN | 1 | 3.6 | missense(1) | 0 | 0 | 0 |
| STK11 | 1 | 3.6 | missense(1) | 0 | 0 |  |
| MTOR | 1 | 3.6 | missense(1) | 0 | 0 | 0 |
| ERBB2 | 1 | 3.6 | missense(1) | 0 | 0 |  |
| MPL | 1 | 3.6 | missense(1) | 0 | 0 | 0 |
| FLT1 | 1 | 3.6 | missense(1) | 0 | 0 | 0 |
| FANCA | 1 | 3.6 | missense(1) | 0 | 0 | 0 |
| CDH2 | 1 | 3.6 | fs(1) | 0 | 0 | 0 |
| FZR1 | 1 | 3.6 | missense(1) | 0 | 0 | 0 |
| FOXL2 | 1 | 3.6 | missense(1) | 0 | 0 | 0 |
| MITF | 1 | 3.6 | missense(1) | 0 | 0 | 0 |
| RUNX1T1 | 1 | 3.6 | missense(1) | 0 | 0 | 0 |
| TLR4 | 1 | 3.6 | missense(1) | 0 | 0 | 0 |
|  |  |  |  |  |  |  |
| MTR | 1 | 3.6 | missense(1) | 0 | 0 | 0 |
| ERCC4 | 1 | 3.6 | missense(1) | 0 | 0 | 0 |
| CHEK2 | 1 | 3.6 | nonsense(1) | 0 | 0 | 0 |
| BRCA2 | 0 | 0 | 0 | 8 | 17.0 | SNV(8) |
| BRCA1 | 0 | 0 | 0 | 6 | 12.8 | SNV(6) |
| EGFR | 0 | 0 | 0 | 4 | 8.5 | SNV(4) |
| KEAP1 | 0 | 0 | 0 | 4 | 8.5 | SNV(4) |
| MET | 0 | 0 | 0 | 3 | 6.4 | SNV(3) |
| BRAF | 0 | 0 | 0 | 2 | 4.3 | SNV(2) |
| CTNNB1 | 0 | 0 | 0 | 2 | 4.3 | SNV(2) |
| CNTNAP2 | 0 | 0 | 0 | 1 | 2.1 | SNV(1) |
| CTNND2 | 0 | 0 | 0 | 1 | 2.1 | SNV(1) |
| IL7R | 0 | 0 | 0 | 1 | 2.1 | SNV(1) |
| EGFR | 0 | 0 | 0 | 0 | 0 |  |
| MET | 0 | 0 | 0 | 0 | 0 |  |
| NRAS | 0 | 0 | 0 | 0 | 0 |  |
| LRP1B | 0 | 0 | 0 | 0 | 0 |  |

**Supplementary Table S11. Results for site prediction (*n* = 28)**

| Predicted site | *n* (%) |
| --- | --- |
| Non–small cell lung cancer | 8 (28.6) |
| Renal cell carcinoma | 6 (21.4) |
| Head and neck squamous carcinoma | 2 (7.1) |
| Hepatocellular carcinoma | 2 (7.1) |
| Ovarian cancer | 2 (7.1) |
| Germinoma | 2 (7.1) |
| Cervical cancer | 2 (7.1) |
| Breast cancer | 1 (3.6) |
| Colon adenocarcinoma | 1 (3.6) |
| Pancreatic adenocarcinoma | 1 (3.6) |
| Urothelial bladder carcinoma | 1 (3.6) |

**Supplementary Table S12. Best overall response for all patients with CUP and the response-evaluable subset according to approval status of ICI monotherapy for predicted primary sites**

|  | All  (*n* = 28) | | RES  (*n* = 23) | |
| --- | --- | --- | --- | --- |
|  | ICI approval for predicted primary site  (*n* = 21) | Other  (*n* = 7) | ICI approval for predicted primary site  (*n* = 17) | Other  (*n* = 6) |
| Objective response rate, *n* (%) | 4 (19.0) | 3 (42.9) | 4 (23.5) | 3 (50.0) |
| 95% CI for % | 5.4–41.9 | 9.9–81.6 | 6.8–49.9 | 11.8–88.2 |
| Best overall response, *n* (%) |  |  |  |  |
| Complete response | 1 (4.8) | 1 (14.3) | 1 (5.9) | 1 (16.7) |
| Partial response | 3 (14.3) | 2 (28.6) | 3 (17.6) | 2 (33.3) |
| Stable disease | 7 (33.3) | 2 (28.6) | 7 (41.2) | 2 (33.3) |
| Progressive disease | 4 (19.0) | 1 (14.3) | 3 (17.6) | 1 (16.7) |
| Non-CR/non-PD | 2 (9.5) | 1 (14.3) |  |  |
| Not evaluated | 4 (19.0) | 0 (0.0) | 3 (17.6) | 0 (0.0) |
| Disease control rate, *n* (%) | 11 (52.4) | 5 (71.4) | 11 (64.7) | 5 (83.3) |
| 95% CI for % | 29.8–74.3 | 29.0–96.3 | 38.3–85.8 | 35.9–99.6 |
| Median PFS (95% CI), months | 6.3 (2.3–18.0) | 8.3 (1.9–NR) | 5.4 (1.6–16.4) | 8.3 (1.9–NR) |
| Median OS (95% CI), months | NR (6.0–NR) | 24.3 (8.9–NR) | 25.1 (5.6–NR) | 24.3 (8.9–NR) |

CI, confidence interval; CR, complete response; CUP, cancer of unknown primary; ICI, immune checkpoint inhibitor; NR, not reached; OS, overall survival; PD, progressive disease; PFS, progression-free survival; RES, response-evaluable subset.
